# Oleic Acid Counters Impaired Blastocyst Development Induced by Palmitic Acid During Mouse Preimplantation Development: Understanding Obesity-Related Declines in Fertility

**DOI:** 10.1007/s43032-020-00223-5

**Published:** 2020-06-15

**Authors:** Maisoon D. Yousif, Michele D. Calder, Jin Tong Du, Kelsey N. Ruetz, Kylie Crocker, Brad L. Urquhart, Dean H. Betts, Basim Abu Rafea, Andrew J. Watson

**Affiliations:** 1 Department of Obstetrics and Gynaecology, The University of Western Ontario, London, Ontario N6A 5C1 Canada; 2 Department of Physiology and Pharmacology, The University of Western Ontario, London, Ontario N6A 5C1 Canada; 3 The Children’s Health Research Institute – Lawson Health Research Institute, London, Ontario N6C 2R5 Canada

**Keywords:** Preimplantation development, Free fatty acids, ER stress, Blastocyst, Fertility

## Abstract

Obesity is associated with altered fatty acid profiles, reduced fertility, and assisted reproductive technology (ART) success. The effects of palmitic acid (PA), oleic acid (OA), and their combination on mouse preimplantation development, endoplasmic reticulum (ER) stress pathway gene expression, lipid droplet formation, and mitochondrial reactive oxygen species (ROS) were characterized. Two-cell stage mouse embryos collected from superovulated and mated CD1 females were placed into culture with KSOMaa medium, or PA alone or in combination with OA for 46 h. PA significantly reduced blastocyst development in a concentration-dependent manner, which was prevented by co-treatment with OA. PA and OA levels in mouse reproductive tracts were assessed by liquid chromatography coupled to mass spectrometry (LC-MS). LC-MS indicated higher concentrations of PA in the mouse oviduct than the uterus. Transcript analysis revealed that PA alone groups had increased ER stress pathway (ATF3, CHOP, and XBP1 splicing) mRNAs, which was alleviated by OA co-treatment. OA co-treatment significantly increased lipid droplet accumulation and significantly decreased mitochondrial ROS from PA treatment alone. PA treatment for only 24 h significantly reduced its impact on blastocyst development from the 2-cell stage. Thus, PA affects ER stress pathway gene expression, lipid droplet accumulation, and mitochondrial ROS in treated preimplantation embryos. These mechanisms may serve to offset free fatty acid exposure effects on preimplantation development, but their protective ability may be overwhelmed by elevated PA.

## Introduction

Global obesity is increasing rapidy [1]. In 2017, Statistics Canada reported that 1.87 million and 1.61 million women of reproductive age (18–49 years old) were overweight or obese, respectively [2]. Obesity increases disease risk and infertility in both males [3] and females [4]. Given that obese women have greater difficulty conceiving, they are more likely to seek fertility treatments and make use of assisted reproductive technologies (ARTs). Unfortunately, studies report substantially poorer outcomes for obese women seeking the assistance of fertility treatments [5–8]. Obese women display decreased responses to gonadotropins used for ovarian stimulation and thus require higher doses of hormones when undergoing ovarian stimulation [7, 9]. With increasing body mass index (BMI), the number of oocytes successfully collected decreases [9, 10]. Thus, regardless of increased gonadotropin doses, obesity is associated with significantly greater risk of unsuccessful ovarian stimulation cycles [7]. In vitro fertilization (IVF) or intracytoplasmic sperm injection (ICSI) in obese women does not affect their ability to become clinically pregnant [8]; however, obese women are at a higher risk for early and recurrent pregnancy loss [9, 11] and miscarriage [8]. During pregnancy, obese women have a higher prevalence of gestational diabetes [11]. Additionally, live birth frequencies are reduced with obesity [7]. The mechanisms underlying the negative direct effects of obesity on reproduction are not fully understood and must be investigated to assist this rapidly growing patient population.

Non-esterified fatty acids (NEFAs) are free fatty acid (FFA) molecules found primarily bound to albumin in circulation [12]. High BMI is associated with elevated NEFA levels in vivo [13]. Palmitic acid (16:0, PA) is the most abundant NEFA in circulation [14], and in the Western diet [15]. Oleic acid (18:1, OA) is the most abundant monounsaturated NEFA in human serum [14]. Concentrations of free PA and OA are between 50 and 70 μM in human follicular fluid and increase in obese women [16]. Although PA concentrations in the human female reproductive tract remain unknown, its presence in serum and follicular fluid suggests that PA and OA are the primary FFAs that embryos are exposed to during preimplantation development. Although PA and OA structurally differ by only two carbons and a double bond, these FFAs have very different metabolic fates [17]. PA, but not OA, is associated with higher diabetes risk [18]. PA displays greater incorporation into phospholipids, and increased lipogenesis, increased beta oxidation, and reduced oxidative phosphorylation than OA does [17].

Exposure to maternal high-fat diet in vivo significantly lowers development of mouse embryos to the blastocyst stage [19]. In mice, transfer of blastocysts from mothers fed a high-fat diet into control normal weight recipients still results in fetal growth restriction and brain developmental abnormalities, indicating that the underlying origin of the problem occurs due to exposure to a high-fat environment during gametogenesis and/or preimplantation development [19]. Increased FFAs in human follicular fluid are correlated with poor cumulus-oocyte-complex (COC) quality [20]. Treatment of bovine COCs with FFAs impairs blastocyst development; however, rescue by the unfolded protein response (UPR)/endoplasmic reticulum (ER) stress pathway inhibitor salubrinal identified UPR activation as a major contributor to FFA-dependent developmental decreases [21]. Studies conducted over the past decade have indicated that the preimplantation embryo is armed with several key stress response mechanisms that, in part, offset the deleterious effects of stress on early development (for review see [22, 23]). Most notably, these are the ER stress pathways, which oversee the unfolded protein response (UPR) and activate stress-responsive gene expression patterns in an attempt to allow the early embryo to effectively adapt to and survive both external and internal stressors [22, 23]. Studies, including ours, have demonstrated the presence and action of ER stress pathways throughout preimplantation development [24–27]. To date, however, studies have not investigated the influences of free fatty acid treatment, i.e., PA and OA on ER stress pathway constituents, and we postulate that differential responses to PA and OA treatment should occur during preimplantation development.

Investigating the direct effects of FFAs on preimplantation embryos in vitro is required to define the mechanistic consequences of exposure to PA and OA, in isolation and in combination, on mammalian preimplantation embryo development in general. We must also learn how the high-fat environment in an obese patient affects preimplantation development. Ultimately, research should inform how the in vitro culture environment can be modified to alleviate deleterious effects of FFA exposure on not only early development but also its longer-term effects on pregnancy, on fetal development, and throughout life. In this study, we report outcomes from experiments in which 2-cell stage mouse embryos were treated with increasing concentrations of PA or OA alone and in combination. We report the effects of these treatments on preimplantation development, ER stress pathway gene expression, lipid droplet formation, and mitochondrial reactive oxygen species (ROS) generation. In addition, we report PA and OA concentrations in the mouse oviduct and uterus. Our outcomes reveal that the PA treatment reduces blastocyst development and alters ER stress pathway transcript levels. OA co-treatment with PA not only reverses PA effects on blastocyst development and ER stress transcript levels but also increases lipid droplet formation and reduces mitochondrial stress. We conclude that preimplantation embryos do employ stress response mechanisms to avoid deleterious effects of PA exposure, but their protective ability may be overwhelmed by exposure to elevated PA.

## Materials and Methods

### Animal Source and Ethics Approval

All experiments were performed using CD-1 mice from Charles River Laboratories (Saint-Constant, QC). All mice were handled according to the Canadian Council on Animal Care and Western University’s Animal Care and Use Policies (protocol #: 2018-075 to Dr. Andrew J. Watson). Mice were housed using conventional housing with a 12-h light/dark cycle and access to standard mouse chow ad libitum.

### Liquid Chromatography Mass Spectrometry Analysis of Oviduct and Uterine PA and OA

Oviduct and uterine concentrations of free PA and OA were quantified using Ultra Performance Liquid Chromatography (UPLC) coupled to quadrupole time of flight (QToF) mass spectrometry. Pairs of ovaries and uterine horns were carefully dissected free of visible fat, placed into cryovials, and frozen over liquid nitrogen before storing at − 80 °C. Following thawing, ovaries or uteri were weighed and 200 μL of acetonitrile containing labeled PA and OA (internal standards, 50 μg/mL each) was added to each sample. Samples were homogenized and incubated on ice for 20 min. Samples were then centrifuged at 20,800×*g*, and the clear supernatant was transferred to a clean glass vial. Analytes were separated using a Phenomenex Phenyl-Hexyl column, 2.1 × 100 mm, 1.7 μ using a Waters Acquity UPLC (Milford, MA) maintained at 40 °C. The mobile phases consisted of water with 0.1% formic acid (A) and acetonitrile with 0.1% formic acid (B) and were pumped across the column at 0.5 mL/min. The gradient was as follows: from 0 to 1.5 min, the gradient went from 80 B to 99% B and was held at 99% B from 1.5 to 2.0 min. The column was reconditioned with 80% B for 1 min before injecting the next sample. The mass spectrometer used was a Waters Xevo G2-S QToF operated in negative ionization mode with the following parameters: capillary voltage, 2 kV; cone voltage, 40 V; source temperature, 150 °C; desolvation temperature, 500 °C; desolvation gas flow, 1000 L/h. Data were acquired using MSe in resolution mode with a scan time of 0.05 s and a mass range of 50–1200 *m*/*z*. The mass accuracy of the instrument was maintained by infusing leucine-enkephalin (1 ng/μL) as the lockspray, which was acquired, every 10 s with a scan time of 0.3 s, which were averaged over 3 scans.

### Mouse Superovulation and Mating

Four- to six-week-old female CD-1 mice were intraperitoneally (IP) injected with 7.5 international units (IU) of pregnant mare’s serum gonadotropin (PMSG, Merck Animal Health, Canada) to stimulate follicular development. Forty-eight hours later, these same females were IP injected with 7.5-IU human chorionic gonadotropin (hCG, Merck Animal Health, Canada) to stimulate ovulation. Immediately after hCG injection, each female was placed in a cage with a single male CD-1 mouse (6–8 months of age) for mating overnight. The following morning, female mice were checked for presence of a seminal plug. Forty-six hours post-injection (hpi) of hCG, female mice were euthanized by CO_2_ asphyxiation (standard operating procedure) and had their oviducts removed and flushed with room temperature M2 flushing medium (Sigma-Aldrich, Oakville, ON) [28–30]. Flushed 2-cell stage embryos were washed 3× in 50-μL drops of potassium simplex optimization medium with amino acids (KSOMaa Evolve, Zenith Biotech, Canada). After washing, embryos were distributed equally among experimental treatment groups and cultured in 20-μL drops under mineral oil (Zenith Biotech, Canada) at a density of 1 embryo per microliter for 46 h under a 5% CO_2_, 5% O_2_, and 90% N_2_ culture atmosphere.

### Free Fatty Acid Preparation and Embryo Culture

Essentially FFA-free bovine serum albumin (BSA, Sigma-Aldrich, Oakville ON) was added to phosphate buffered saline (PBS) and dissolved overnight to create a 20% BSA solution. This solution was filter sterilized and used downstream for conjugation to PA (Sigma-Aldrich, Oakville ON) or OA (Sigma-Aldrich, Oakville, ON). Stock PA and OA solutions were prepared by solubilizing each FFA in RNAse-free water and NaOH at 70 °C to create a 20-mM solution. This stock sample was conjugated in a 2:1 molar ratio to BSA to create a final 500-μM FFA solution that was stored at 4 °C. Experiments were performed where 2-cell stage embryos were cultured in FFA conjugated 2:1 to BSA in KSOMaa.

### PA and OA Concentration Response and Treatment Experiments

We first defined the concentration effects of PA and OA treatments alone on development of 2-cell stage embryos to the blastocyst stage. The treatment concentration series for PA consisted of 25, 50, and 100 μM, and for OA was 50, 100, 250, and 500 μM. For each experimental replicate and treatment group, twenty 2-cell embryos were placed into 20-μL drops of the appropriate treatment medium covered by embryo-grade mineral oil (Zenith Biotech, Canada). Embryos were cultured under a 5% CO_2_, 5% O_2_, and 90% N_2_ atmosphere at 37 °C for 46 h to assess progression to the blastocyst stage.

Once effects of single PA and OA treatment on development was characterized, we proceeded with conducting single treatment and PA and OA combination treatment experiments. Treatments included control (+ 1.5% BSA); 100-μM PA alone; or 100-μM PA with either 50-; 100-, or 250-μM OA in combination in KSOMaa. The BSA content was controlled between treatments using a 1.5% BSA in KSOMaa control, which was prepared from the original 20% BSA solution. For each experimental replicate and treatment group, twenty 2-cell embryos were placed into 20-μL drops of the appropriate treatment medium covered by embryo-grade mineral oil (Zenith Biotech, Canada). Embryos were cultured under a 5% CO_2_, 5% O_2_, and 90% N_2_ atmosphere at 37 °C for 46 h to assess progression to the blastocyst stage.

To assess the effect of PA treatment time on development to the blastocyst stage, twenty 2-cell embryos were placed in control media or 100-μM PA treatment media for 24-h culture, and then embryos were washed and placed into PA-free control medium for either 24 or 48 additional hours of culture, leaving the embryos in culture for a total of 48 and 72 h, respectively. Blastocyst development was assessed at the end of the 48- and 72-h culture periods.

### Developmental Stage Analysis

At the end of each experimental replicate culture period, embryos were examined under a light-dissecting microscope. The proportion of embryos at each stage of preimplantation development was recorded. Embryos were classified as either a blastocyst, morula (compacted embryo), 8-cell, 4-cell, 2-cell, or degenerated embryo. Morulae were defined as embryos where distinct blastomeres could not be identified or counted, but no visible fluid-filled cavity was apparent. Blastocysts were defined as any embryo with a visible fluid-filled cavity. After developmental analysis, embryos were either snap frozen at − 80 °C for RNA extraction or fixed for future staining and confocal microscopy.

### RNA Extraction and Reverse Transcription

The ARCTURUS PicoPure RNA Isolation Kit (Life Technologies, Burlington, ON) was used per the manufacturer protocol to extract total RNA from pools of 20 preimplantation mouse embryos in each sample and replicate [30]. Exogenous luciferase mRNA (0.025 pg/embryo, Promega, USA) was added for use as a reference standard gene when analyzing mRNA quantities using the delta delta cycle threshold (2^-△△Ct^) method. To eliminate genomic DNA, DNase 1 (RNA-free DNase kit, Qiagen, Louisville, KY) was added in an additional step. The VILO cDNA Synthesis Kit (Invitrogen, Burlington, ON) was used according to the manufacturer protocol to reverse transcribe the extracted RNA into cDNA. cDNA was diluted to 1 embryo/μL in PCR-grade water.

### XBP1 Spliced and Unspliced Transcript Detection

To confirm the effectiveness/quality of cDNA synthesis, polymerase chain reaction (PCR) was performed for H2A and luciferase on each embryo reverse transcribed sample. Each reaction included 2.5 μL of 10X PCR buffer, 0.75-μL MgCl_2_, 0.5-μL dNTPs, 0.1 μL each of the forward and reverse primer, 0.2-μL Taq polymerase, and 19.85-μL RNase-free water. The cycling conditions for both H2A and luciferase were 94 °C for 2 min, followed by 44 cycles of 94 °C for 30 s, 61 °C (H2A) or 59 °C (luciferase) for 30 s, 72 °C for 1 min, and a last step of 72 °C for 10 min. PCR products were run on a 2% agarose gel for H2A/luciferase with ethidium bromide in TAE running buffer at 100 V for 1 h, after which the gel was imaged. For XBP1 mRNA transcript detection, cycling conditions were 94 °C for 2 min, followed by 45 cycles of 94 °C for 30 s, 61 °C for 30 s, 72 °C for 1 min, and a last step of 72 °C for 10 min. XBP1 PCR products were run on a 4% agarose gel with TBE running buffer at 60 V for 3 h. Positive controls contained cDNA from embryos that were treated with the potent and reliable ER stress inducer tunicamyin [31], while negative controls included RNase-free water in place of cDNA. Splicing of XBP1 was quantified using the ImageJ software on three biological replicates.

### Quantitative RT-PCR Assessment of ATF3, CHOP, and ATF6, Transcripts

Quantitative RT-PCR was performed using Taqman primer probes (Invitrogen, Canada) for target genes in a 384-well plate. Master mix (MM) of TaqMan Gene Expression Mastermix (volume = 0.5 × total volume (V)), TaqMan primer probe (volume = 0.05 × V), and RNase-free water (volume = 0.4 × V) were prepared and 62.7 μL aliquoted into Eppendorf tubes, to which 3.3 μL (equivalent to 1 embryo/μL) of embryo cDNA was added. This mixture of master mix and cDNA was vortexed, and 20 μL was added in triplicate wells to the PCR plate. For each experimental series, a negative no transcript control was included, where RNase-free water replaced the cDNA. Using a BioRad CFX384 Touch™ Real-Time PCR Detection System, quantitative RT-PCR (qPCR) was run according to the following conditions: 5 min at 95 °C (AmpliTaq GOLD DNA polymerase activation step), followed by 50 cycles of 15 s at 95 °C (denaturation) and 1 min at 60 °C (annealing and extension). To assess ER stress, primer probes for ATF3 (Mm00476032_m1), CHOP (Mm01135937_g1 Ddit3), and ATF6 (Mm01295317_m1 Atf6) were used. Three biological replicates were assessed for each gene, for each treatment, and for each experimental replicate.

### BODIPY 493/503 Staining to Assess Embryo Lipid Droplet Accumulation

After the 46-h culture treatment period, embryos from treatment and control were placed in 2% paraformaldehyde in PBS for 30 min for fixing. They were then washed 3X in PBS and stored at 4 °C. BODIPY (ThermoFisher Scientific, Mississauga, ON) was solubilized in DMSO to a concentration of 2.5 mg/mL to create a stock solution, and an aliquot was diluted to 20 μg/mL using the KSOMaa medium. Embryos from all treatment groups, except the negative control, were placed in 20-μL drops of BODIPY solution covered in embryo-grade mineral oil for 1 h at room temperature. Embryos were then washed 3× in PBS and added to 10-μL drops of PBS covered in mineral oil in a glass-bottom dish. An independent group of embryos exposed to OA at varying concentrations was used to set the laser intensity (Alexa Fluor 488). Embryos were imaged using confocal microscopy (Zeiss LSM800 microscope) at × 10 magnification. Z-stacks were imaged in 5-μm slices. Images were processed using the FIJI and Ilastik software [32]. Within a group, each embryo was defined as a biological replicate. The fluorescence intensity of each individual embryo in each treatment group was quantified to determine mean fluorescence within a treatment group using the Ilastik software.

### MitoSox™ Red Superoxide Stain

MitoSox™ Red mitochondrial superoxide indicator (Life Technologies, Burlington, ON) was solubilized in DMSO and diluted using KSOMaa to a concentration of 5 μM. Culture dishes were prepared with 20-μL drops of this solution covered in embryo-grade mineral oil. Live embryos were incubated in this solution for 1 h at 37 °C, 5% O_2_, 5% CO_2_, and 90% N_2_. A no-MitoSox™ Red negative control was also included. Embryos were then washed 3× in KSOMaa and added to 10-μL drops of KSOMaa in a glass-bottom dish for imaging by confocal microscopy (Zeiss LSM800 microscope) at × 10 magnification. An independent group of embryos exposed to 100-μM PA treatment was used to set the confocal laser intensity (Alexa Fluor 568). Z-stacks were imaged in 5-μm slices. Images were processed using the FIJI and Ilastik software [32]. Within a group, each embryo was a biological replicate. The fluorescence intensity of each individual embryo in each treatment group was quantified to determine mean fluorescence within a treatment group using the Ilastik software.

### Statistical Analyses

GraphPad PRISM 8 (https://www.graphpad.com/scientific-software/prism/) was employed to perform statistical analyses. For analysis of tissue concentrations of PA and OA by mass spectrometry, a two-way analysis of variance (ANOVA) was performed with main factors of age and tissue type and means compared by using Tukey’s multiple comparisons tests. For all subsequent experiments, a minimum of three biological replicates was conducted. For imaging studies, a biological replicate was defined as a single embryo within a treatment. However, for assessments of developmental stage and mRNA abundance through qPCR, a biological replicate consisted of a single pool of 20 embryos within a treatment group. Blastocyst development was analyzed using a one-way ANOVA test, and means were compared to one another by an ad hoc Tukey’s multiple comparisons test. The same statistical analyses were used when analyzing the number of embryos arrested at different stages of development with the FFA treatments. Using cycle threshold values from qRT-PCR, relative amounts of mRNA were quantified using the delta delta cycle threshold (2^-△△Ct^) method followed by one-way ANOVA and Tukey’s multiple comparisons tests. Data analysis for BODIPY and MitoSox™ Red outcomes included applying one-way ANOVA with Tukey’s multiple comparisons tests. For all tests, *p* values of less than or equal to 0.05 were considered significantly different from one another.

## Results

### PA and OA Concentrations in Mouse Oviduct and Uterus

Table 1 shows the mean ± standard error (SE) of the mean concentrations of PA and OA that were detected using liquid chromatography mass spectrometry (LC-MS) in whole mouse oviduct (4-week-old mice, *n* = 9; 8-month-old mice, *n* = 12) and uterine (4-week-old mice, *n* = 10; 8-month-old mice, *n* = 12) lysates. Values were corrected for tissue weight; pairs of oviducts ranged from 5.1 to 13.3 mg, while uteri ranged from 40.1 to 142.1 mg. Older mice displayed higher oviduct and uterine weights than 4-week-old mice. In the two-way ANOVA for PA, there was no effect of age or interaction; however, there was a significant effect of tissue type. Oviducts had significantly higher (*p* < 0.001) concentrations of PA than uteri. PA concentrations were significantly higher (*p* < 0.05) than OA concentrations in oviducts and uteri for both age groups. In contrast, for OA, there was a significant interaction of age by tissue (*p* < 0.02), such that young oviducts had lower OA concentrations than older oviducts, or young or older uteri. There were also significant effects of age (*p* < 0.01) and tissue (*p* < 0.001) with uteri having higher average OA concentrations compared with oviducts (Table 1).

**Table 1:** Palmitic acid and oleic acid concentrations in mouse oviduct and uterus

	Palmitic acid (μM)		Oleic acid (μM)	
Mouse groups	Oviduct	Uterus	Oviduct	Uterus
4-week CD1	377.6 ± 33.2^(9)^	192.8 ± 21.6^(10)^	18.0 ± 4.4^(9)c^	51.0 ± 6.6^(10)d^
8-month CD1	404.9 ± 33.2^(12)^	145.1 ± 8.9^(12)^	44.3 ± 3.2^(12)d^	51.8 ± 4.2^(12)d^
Combined values	393.2 ± 23.3^(21)a*^	166.8 ± 11.9^(22)b§^	33.0 ± 2.4^(21)*^	51.4 ± 3.7^(22)§^

N values in brackets for each group

All values represent mean ± SE

^a,b^Within PA, superscripts denote significant differences between tissues, *p* < 0.001

^c,d^Within OA, superscripts denote an age by tissue interaction, *p* < 0.02

^*^Within oviducts, PA is higher than OA, *p* < 0.001

^§^Within uteri, PA is higher than OA, *p* < 0.001

### Concentration-Responsive Effects of PA and OA Treatment on Mouse Preimplantation Development

**Fig. 1:**
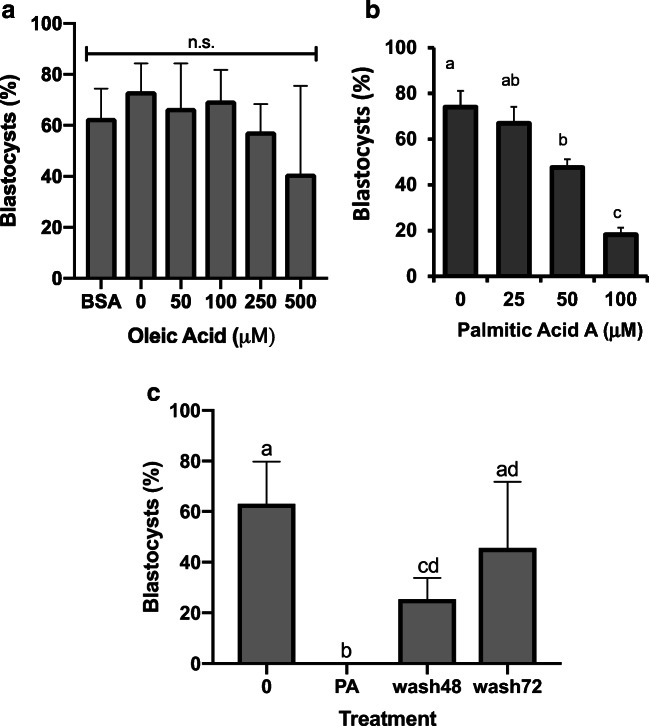
Concentration-responsive effects of PA and OA on blastocyst development. The percentage of blastocysts after 46-h culture in increasing concentrations of **a** OA or **b** PA. Treatment of 2-cell embryos in 1.5% BSA and at all OA doses did not significantly affect development to the blastocyst stage (**a**, *p* > 0.05, *n* = 3). In contrast, treatment with concentrations of PA over 25 μM resulted in a significant decline in development to the blastocyst stage (**b**, *p* < 0.05, *n* = 3). **c** Effects of 24-h treatment with 100-μM PA followed by culture in PA-free control medium for 24 h (wash48 h) and 48 h (wash72 h) after treatment on development from the 2-cell stage. Twenty-four-hour PA treatment (wash48) at the 2-cell stage did not reduce blastocyst development as severely as 46-h PA treatment **(**PA, **c**, *n* = 3–8). In fact, 2 cell embryos treated for 24 h with 100-μM PA and then placed into control medium for 48 h (wash72) displayed an equivalent level of development to the blastocyst stage as untreated controls (**c**). Bars represent the mean percentage of embryos at the blastocyst stage + standard deviation (SD) (**a**, **b**, **c**). Bars with *p* values < 0.05 are statistically significant from one another

**Fig. 2:**
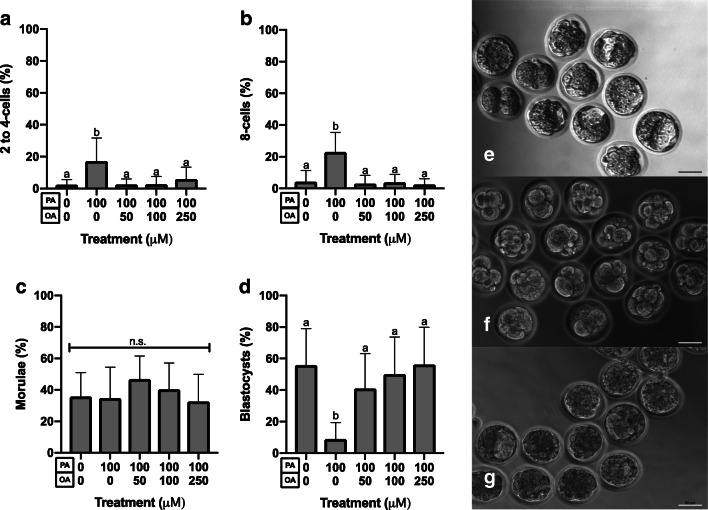
PA and OA co-treatment effects on blastocyst development. 2-cell stage embryos were placed into culture in either control medium (*n* = 13); 100-μM PA alone (*n* = 13); or 100-μM PA in combination with 50- (*n* = 10), 100- (*n* = 11), or 250- (*n* = 12) μM OA. PA alone significantly reduced (*p* < 0.05) development to the blastocyst stage (**d**), with a corresponding significant increase in the proportion of early cleavage-stage embryos undergoing a developmental arrest before the morula stage (**a**–**c**). The proportion of morula did not differ significantly between treatments (**c**). OA co-treatment at all doses (50–250-μM concentrations; **d**) alleviated the significant decline in development of PA-treated 2-cell stage embryos to the blastocyst stage. Treatment with 100-μM PA increased the proportion of embryos displaying cell fragmentation and unequal blastomeres relative to controls (**e** controls; **f** PA treated). Co-culture of 2-cell embryos in 100-μM PA with OA resulted in improved embryo morphology (**g**). Bars represent the mean percentage of embryos at each stage + SD. Bars with different letters are statistically significant from one another. Scale bars are equal to 50 μm

The concentration-responsive effects of treating 2-cell stage mouse embryos with increasing doses of PA and OA are shown in Fig. 1. OA concentration, at all doses (46 h of treatment), did not have a significant (*p* > 0.05) effect on the progression of treated embryos to the blastocyst stage (Fig. 1a). PA treatment in contrast resulted in a significant decline (*p* < 0.05) in the progression to the blastocyst stage for concentrations over 25 μM (Fig. 1b). This result was accompanied by a corresponding increase in the proportion of early cleavage-stage embryos observed in PA treatment concentrations greater than 50 μM (data not shown). Embryos treated with PA dose higher than 50 μM increasingly displayed cell fragmentation and unequal blastomere size (Fig. 2f). However, embryos exposed to OA alone displayed normal morphological characteristics expected for mouse preimplantation embryos at all stages (data not shown). In contrast, 2-cell embryos treated with 100-μM PA for 24 h only and then placed into PA-free control medium for 24 h (for a total of 48 h of culture) displayed significantly (*p* < 0.05) higher development to the blastocyst stage than that displayed by 46-h PA-treated 2-cell embryos (Fig. 1c, wash48). Furthermore, when 24-h PA-treated 2-cell embryos were cultured in normal control medium for 48 h after treatment (for a total treatment and culture time of 72 h), blastocyst development was no longer significantly different from control levels of development (Fig. 1c, wash72). Since 100-μM PA-treated embryos displayed a capacity to recover from treatment, we adopted this PA concentration level as standard for all subsequent experiments. Controls for BSA content (BSA = 1.5%; 0 = 1 mg/ml BSA, Fig. 1a) demonstrated that the BSA content of the medium did not adversely affect development of treated 2-cell embryos to the blastocyst stage.

### Effect of PA and OA Co-Treatment on Mouse Preimplantation Development

To characterize the effects of combined treatment, 2-cell stage embryos were placed into culture in either control medium; medium with 100-μM PA alone; or medium (100-μM PA) in combination with 50-, 100-, or 250-μM OA for 46 h. PA alone significantly reduced development to the blastocyst stage (*p* < 0.05, Fig. 2d), with a corresponding significant increase in the proportion of early cleavage-stage embryos undergoing a developmental arrest before the morula stage (*p* < 0.05, Fig. 2a–c). The proportion of morula-stage embryos did not differ between treatments (*p* > 0.05, Fig. 2c). In complete contrast to the PA alone treatment results, OA co-treatment at all doses (50–250-μM concentrations (*p* < 0.05, Fig. 2d)) offsets the PA-induced significant decline in the development of 2-cell stage embryos to the blastocyst stage in vitro. Figure 2f displays the typical morphology of embryos treated with 100-μM PA compared with untreated controls (Fig. 2e) and to that of 2-cell stage embryos co-cultured in 100-μM PA with all OA concentrations (Fig. 2g, PA+OA).

### Effects of PA and OA on Preimplantation Embryo ER Stress Pathway Transcripts

**Fig. 3:**
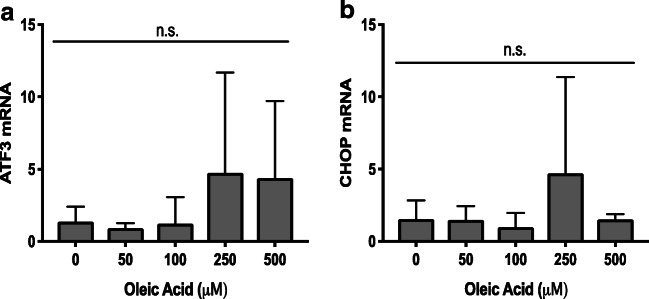
Effect of OA treatment on ER stress transcripts. The effects of 50–500-μM OA treatment alone for 46 h on ATF3 and CHOP transcript levels in mouse blastocysts. ATF3 and CHOP mRNA levels were not significantly affected by OA treatment at any dose (**a**, **b**, *p* > 0.05. *n* = 3). Bars represent mean fold change in mRNA abundance relative to the control + SD

**Fig. 4:**
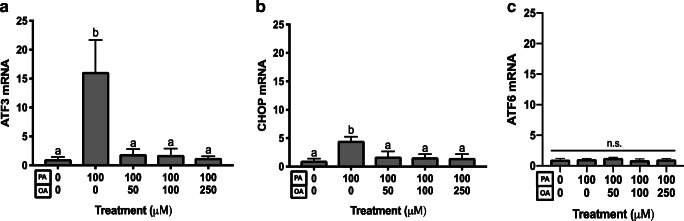
Effect of PA and OA co-treatment on ER stress transcripts. The effects of exposure to 100-μM PA treatment alone, and PA and OA combination treatments for 46 h on transcript levels of marker mRNAs representing ER stress pathways were assessed. In 100-μM PA-treated embryos, ATF3 transcript abundance increased significantly (*p* < 0.05, *n* = 3) to 15 fold the control levels, and CHOP transcript increased significantly (*p* < 0.05, *n* = 3) to 5 fold the control levels (**a**, **b).** OA co-treatment at all doses alleviated the PA-induced increases in CHOP and ATF3 mRNA levels (**a**, **b**, *p* < 0.05, *n* = 3). PA treatment (100 μM) alone or in combination with OA did not significantly affect the relative levels of ATF6 mRNAs across all groups (**c**, *p* > 0.05, *n* = 3). Bars represent mean fold change in mRNA abundance relative to the control + SD. Bars with different letters are statistically significant from one another

**Fig. 5:**
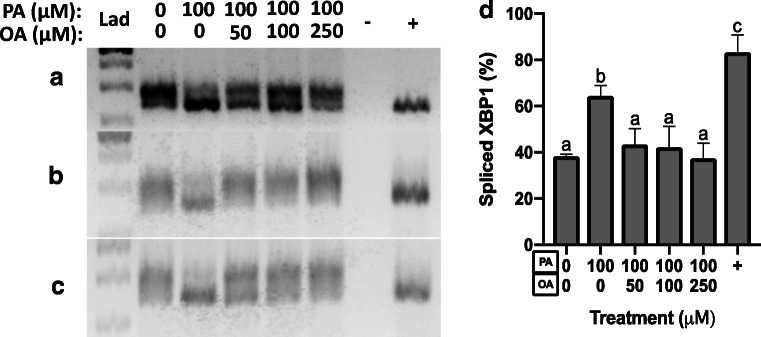
Effect of PA and OA co-treatment on XBP1 splicing. To characterize effects to the IRE1 arm of the ER stress pathway, we measured effects of PA alone or with OA on the relative levels of spliced XBP1 mRNA. Treatment with 100-μM PA significantly (*p* < 0.05, *n* = 3) increased XBP1 mRNA splicing (A–D) relative to controls. XBP1 mRNA splicing was significantly reduced (*p* < 0.05, *n* = 3) when co-treatment with OA occurred (A–D). Controls included a no-cDNA negative control (−) and tunicamycin-treated embryo positive control (+). A–C represent three biological replicates. Bars in D represent mean percentage of spliced XBP1 transcripts + SD. Bars with different letters are statistically significant from one another

We next characterized the effects of PA and OA treatment on preimplantation development ER stress pathways. To do this, we characterized the effect of 100-μM PA treatment alone and PA and OA combination treatment on transcript levels of markers representing all three main ER stress pathways. ATF3 and CHOP mRNA levels were not significantly affected by OA treatment at any dose (Fig. 3a and b). However, in embryos treated with 100-μM PA alone, ATF3 transcript abundance increased significantly (*p* < 0.05) to over 15 fold that of control, and CHOP transcript abundance was also significantly (*p* < 0.05) increased and approached 5-fold greater levels than controls (Fig. 4a and b). In contrast, OA co-treatment at all doses alleviated the PA-induced increase in CHOP and ATF3 mRNA levels (Fig. 4a and b). Interestingly, PA treatment (100 μM) alone or in combination with OA did not significantly affect the relative levels of ATF6 mRNAs across all groups, suggesting that this arm of the UPR may not be influenced by FFA exposure during mouse preimplantation development (Fig. 4c). Investigation of the IRE1 arm of the ER stress pathway required us to measure changes in the proportion of XBP1 spliced and unspliced mRNAs. XBP1 is downstream of IRE1 pathway activation, and its activation is dependent upon transcript splicing, with spliced transcript representing activated XBP1 that serves as a nuclear transcription factor upon activation of ER stress response. Treatment with 100-μM PA significantly (*p* < 0.05) increased in XBP1 mRNA splicing (Fig. 5A–C, three separate experimental replicates displayed) relative to controls. In contrast, XBP1 mRNA splicing was significantly reduced (*p* < 0.05) when co-treatment with all OA concentrations was employed (Fig. 5D), while tunicamycin (an ER stress activator)-treated positive (+) controls displayed a significant increase in spliced XBP1 (Fig. 5D).

### Effects of OA and PA Treatment on Preimplantation Embryo Lipid Droplet Abundance

**Fig. 6:**
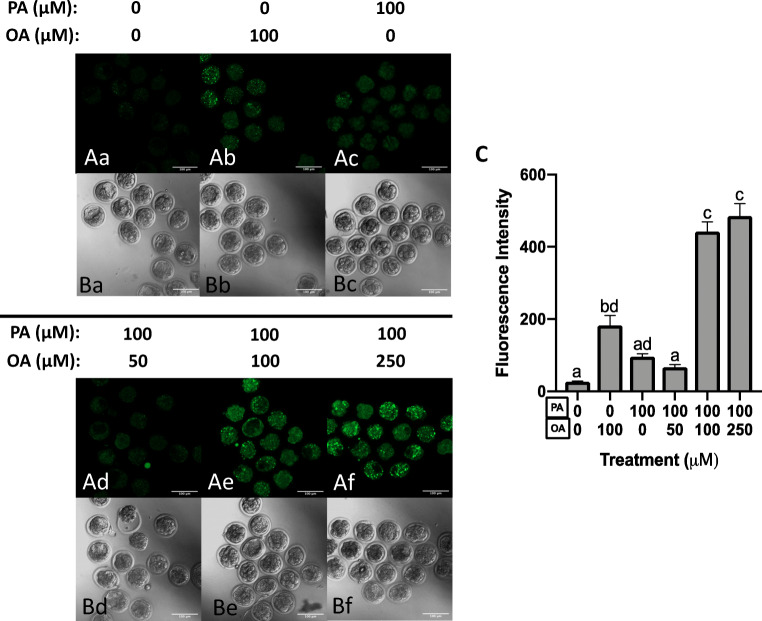
OA treatment increases lipid droplet levels. Compared with control media (*n* = 14), OA treatment alone (100 μM, *n* = 12) significantly increased (*p* < 0.05, Ab, Bb, C) LD content. PA treatment (100 μM, *n* = 18) alone did not significantly affect (*p* > 0.05, Ac, Bc, C) LD content. Co-treatment with 50-μM OA (*n* = 13, Ad, Bd, C) did not significantly alter LD levels from control (Aa, Ba, C) or PA alone (Ac, Bc, C). OA at 100 (*n* = 15) and 250 (*n* = 16) μM significantly increased (*p* < 0.05, Ae, Be, Af, Bf, C) LD levels 16 and 18 times greater than controls, respectively. Scale bars represent 100 μm. Bars in C represent mean BODIPY 493/503 fluorescence per embryo + standard error (SE). Data is presented in fluorescence units with background fluorescence subtracted. Bars with different letters are statistically significant from one another

Next, the influence of FFA treatment on lipid droplet (LD) accumulation in mouse preimplantation embryos was characterized. Relative to embryos exposed to control media, OA treatment alone (100 μM) significantly increased (*p* < 0.05, Fig. 6Ab, Bb, and C) LD content. However, PA treatment (100 μM) alone did not significantly affect LD content (*p* > 0.05, Fig. 6Ac, Bc, and C). Co-treatment with 50-μM OA (Fig. 6Ad, Bd, and C) also did not significantly alter LD levels from that observed for controls (Fig. 6Aa, Ba, and C), PA alone (Fig. 6Ac, Bc, and C), or 100-μM OA-treated embryos (Fig. 6Ab, Bb, and C). However, OA concentrations of 100 and 250 μM in combination with PA (100 μM) significantly increased (*p* < 0.05, Fig. 6Ae, Be, Af, Bf, and C) LD levels to 16 and 18 times greater than those of controls, respectively.

### Effects of OA and PA Treatment on Preimplantation Embryo on Mitochondrial Superoxide Levels

**Fig. 7:**
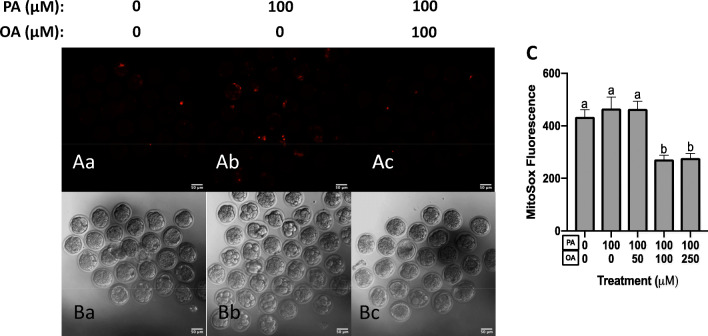
OA reduces MitoSox Red Fluorescence. Mitochondrial superoxide produced by embryos exposed to PA treatment alone (100 μM, *n* = 28) (Ab, Bb, C) or in combination with 50-μM OA (*n* = 24, images not shown) did not differ significantly from untreated control embryos (*n* = 19, Aa, Ba, C). Mitochondrial superoxide levels were significantly lower in embryos exposed to 100-μM PA in combination with 100-μM (*n* = 24, Ac, Bc, C) or 250-μM OA (*n* = 24, images not shown). Bars in C represent mean MitoSox Red fluorescence per embryo + standard error (SE). Data are presented in fluorescence units with background fluorescence subtracted. Bars with different letters are statistically significant from one another. Scale bars represent 50 μm

We next characterized whether PA (100 μM) or OA co-treatment affected preimplantation mouse embryonic mitochondrial superoxide levels, by measuring MitoSox™ Red fluorescence. Mitochondrial superoxide produced by embryos exposed to 100-μM PA treatment alone (Fig. 7Ab, Bb, and C) or in combination with 50-μM OA (images not shown, Fig. 7C) did not differ significantly from untreated control embryos (Fig. 7Aa, Ba, and C). Interestingly, we did observe that mitochondrial superoxide levels were significantly lower (*p* < 0.05) in embryos exposed to 100-μM PA in combination with 100-μM or 250-μM OA (Fig. 7Ac, Bc, and C). Thus, OA treatment displayed an ability to reduce basal levels and mitochondrial superoxide levels within in vitro cultured embryos.

## Discussion

We have discovered that mouse oviductal PA concentrations are nearly twice as high as mouse uterine PA concentrations and are much higher than OA concentrations in these tissues. Treatment of 2-cell stage mouse embryos in vitro alone with PA at lower than reproductive tract tissue concentrations disrupts preimplantation development to the blastocyst stage and is accompanied by elevated ER stress pathway transcript levels. In contrast, treatment with OA up to 500 μM does not significantly affect development of 2-cell stage mouse embryos to the blastocyst stage; however, it increases LD accumulation and reduces mitochondrial ROS in early embryos. Most importantly, the addition of OA as a co-treatment with PA counters the negative developmental impact of PA alone as well as its impact on ER stress transcript levels. Collectively, our results suggest that mouse preimplantation embryos are exquisitely sensitive to external FFA levels, especially for PA. The early embryo appears to respond to this stress, as it does for many other stressors, in part, by upregulating the ER stress pathways. However, PA levels may exceed this adaptive capacity, resulting in impaired preimplantation development. Our results are especially poignant for obese patients seeking fertility treatment, as they bring into focus the possibility of an obesity-conditioned reproductive tract actively suppressing preimplantation development and consequently pregnancy. Research must identify approaches in which the debilitating effects of PA exposure may be alleviated.

The literature has established that PA and OA have differential effects in many tissues. PA is generally viewed as being pro-apoptotic, while OA has beneficial and protective effects against PA [15, 33, 34]. For example, a recent review reported that PA is pro-inflammatory and induces ER stress [35]. However, the negative effects of PA can be overcome by relatively lower concentrations of OA in many tissues, including hepatocytes and pancreatic β cells [35]. PA reduces insulin sensitivity in hepatocytes [15] and skeletal muscle [36], and thereby contributes to the development of type 2 diabetes mellitus (T2DM). Conversely, OA is protective against insulin resistance and T2DM [35]. PA dose-dependently increased podocyte ROS production and apoptosis, suggesting its contribution to podocyte loss in the kidney and subsequent development of diabetic nephropathy [37]. When administered intraperitoneally, PA is linked to behavioral abnormalities, such as anxiety-like symptoms in mice [38]. Studies indicate that PA exerts its negative effects through a variety of mechanisms, including alterations to cellular membranes [39], ER stress [34, 40–46], diacylglycerol (DAG) accumulation [47], mitochondrial dysfunction, and ROS production [48, 49]. These effects, which are collectively called “lipotoxicity,” ultimately result in ER stress and subsequent apoptosis if the stress is not alleviated.

ER stress occurs in response to an accumulation of unfolded or misfolded proteins in the ER. The UPR pathway is activated in an attempt to alleviate this stress [50]. If unable to establish homeostasis, the UPR results in apoptosis [51]. Differential effects of physiologically relevant doses of PA and OA on ER stress were observed in hepatoma cells, where PA induced ER stress and OA did not [42, 52]. In primary neonatal cardiomyocytes, PA induced both ER stress and apoptosis, whereas OA alone did not [34]. In rat liver hepatoma cells, 500-μM PA treatment induced ER stress and elevated PERK and IRE1α phosphorylation, and XBP1 splicing upon PA exposure [42]. Given the close relationship in other tissues between PA exposure and ER stress activation [34, 35, 41–47], we assessed the UPR and ER stress pathways in our embryos using RT-PCR for XBP1 splicing and quantitative RT-PCR for CHOP, ATF3, and ATF6. Thus, in total, we investigated the effects of PA and OA treatment on all three arms of the UPR, with ATF3 and CHOP falling under the PERK arm, XBP1 splicing a marker of the IRE1α arm, and ATF6 falling under its own arm. As is consistent with previous studies [24–27, 34, 53], PA altered ER stress pathway transcripts but OA did not. However, a key limitation of our outcomes, is that UPR and ER stress pathway members are regulated via posttranslational modifications, such as phosphorylation of PERK, IRE1, and ATF6. Protein phosphorylation can be assessed through western blotting; however, the low protein abundance in mouse preimplantation embryos makes these experiments technically challenging to successfully perform and requires large embryo pools to effectively apply. In addition, we cannot conclude that the changes we have observed “cause” the developmental blockade observed following PA treatment. We are simply reporting key observations made in embryo pools following 46 h of treatment. We believe that these observations are the first to indicate that exposure to elevated PA may affect ER stress pathway constituent expression and that the addition of OA significantly reverses that occurrence. There are many more experimental designs and time course experiments that must be conducted to fully understand this outcome, and we are very interested in pursuing outcomes to those stage-specific effects in future experiments. Our results are consistent with the growing conclusion that OA is protective against PA’s effects, specifically in that it alleviates PA-induced ER stress in mouse preimplantation embryos [22–27, 34].

A recent review by Fayezi et al. [54] outlined the most likely mechanisms through which OA exerts its protective effects. These included but were not limited to 1) altering triacylglycerol (TAG) synthesis, 2) reducing oxidative stress, and 3) altering energy production. Fatty acids (FAs), either bound to albumin or contained in lipoproteins, are found extracellularly. Once released, FAs enter the cell where they are conjugated to CoA [55]. A series of enzymatically catalyzed reactions in the ER allows for the formation of DAGs, which can then form TAGs [55, 56]. Conversion of DAG to TAG is the final step of TAG formation and is catalyzed by diacylglycerol acyltransferase (DGAT) enzymes DGAT1 and DGAT2 [56]. PA and OA differentially affect LD accumulation. Since OA is steatotic and causes lipid accumulation, exposure to OA increases LD number [47, 54]. LDs are made up of a phospholipid monolayer surrounding a neutral lipid core mainly consisting of TAGs [55]. Piccolis et al. [44] reported that when human leukemia cells were exposed to PA, a primary mechanism responsible for lipotoxicity was ER stress, which was associated with an accumulation of PA and DAGs in the cells. In cardiomyoblasts, PA treatment reduced LD levels, whereas OA increased LD accumulation [47]. This decrease in LDs with PA treatment was not due to a lower supply of neutral lipid, as more PA accumulated in the cells than OA, and fatty acid oxidation was reduced with PA exposure [47]. The investigators concluded that the reductions in LDs with PA were due to PA storage as DAGs, whereas OA is largely stored as TAGs [47]. Accumulation of PA in the ER likely contributes to induction of ER stress, activation of the UPR, and apoptosis should the ER stress not be relieved. In our study, OA, but not PA, induced lipid droplet accumulation in mouse preimplantation embryos. Inclusion of OA concentrations equal to or higher than the 100-μM PA in co-culture resulted in lipid droplet accumulation that was significantly higher than control, PA-only, or OA-only treated embryos. When quantified, there was no difference in BODIPY fluorescence in PA-treated embryo from controls. This outcome seems reasonable, as we suggest the PA could be accumulating as DAGs and would not accumulate within LDs. Collectively, our findings suggest that OA directs accumulation of TAGs within cytoplasmic lipid droplets during mouse preimplantation development. Given the similarity of our outcomes to studies applied to other tissues, we suggest that there is reasonable likelihood for common mechanistic events arising during preimplantation development.

Studies have established that PA treatment often results in mitochondrial dysfunction [57–62]. To consider this effect in preimplantation embryos, mitochondrial superoxide levels in embryos exposed to PA alone or in combination with OA were quantified. When OA was added to skeletal myotubes cultured in PA, the PA-induced increase in MitoSox Red fluorescence was dampened. Interestingly, in our study, the embryos cultured in control, PA alone, or PA co-cultured with 50-μM OA did not vary in the levels of detectable mitochondrial superoxide. Compared with these groups, embryos co-cultured with PA and 100- or 250-μM OA produced significantly less mitochondrial superoxide. We propose that the 100-μM PA treatment we employed was not enough to elevate mitochondrial ROS. Clearly, however, elevated OA concentrations (100 or 250 μM) have the capacity to reduce mitochondrial ROS levels even below that of basal culture-induced levels. Thus, it is possible that adding OA alone to the culture medium may be beneficial for supporting preimplantation embryo mitochondrial function. However, the differences in mitochondrial superoxide levels may not be indicative of mitochondrial superoxide production.

Overall, our outcomes essentially mirror those observed regarding PA and OA’s effects across many different tissues; however, the effect of PA and OA in the reproductive tract, specifically during preimplantation embryo development, has not been extensively investigated and defined. The timing of preimplantation embryo development is a key measure of embryo developmental competence [62]. Developmental competence is also often assessed by embryo cell number [62]. Although we did not measure blastocyst cell number, the fact that embryos exposed to OA alone or in combination with PA were able to reach the blastocyst stage within a normal culture period is indicative of developmental competence and likely capacity to initiate a pregnancy. The significantly decreased progression throughout preimplantation development that we observed following PA treatment is an ominous sign and is highly suggestive of decreased embryo viability even if the blastocyst stage is reached. Additionally, morphological assessment of PA-treated embryos displayed uneven blastomeres and cell fragmentation, both of which are signs of reduced embryo developmental competence [62, 63]. These morphological changes did not occur with PA and OA co-treatment, suggesting that mouse embryos that were exposed to OA may have greater adaptive ability. When PA treatment time was reduced to 24 h, we observed normal development to the blastocyst stage following 48 h of post-treatment culture in control medium. This indicates that 24-h exposure to PA delayed the embryos’ development; however, removal of PA and additional culture time allowed the embryos to overcome this delay. We believe this to be an important finding, as it suggests that removing embryos from a high fatty acid environment may improve their development. Further research must be conducted to investigate the underlying mechanisms controlling the developmental blockade resulting from PA treatment.

In conclusion our outcomes have revealed that PA treatment reduces blastocyst development and alters ER stress pathway transcript levels. OA co-treatment with PA not only limits PA effects on blastocyst development and ER stress transcript levels but also increases lipid droplet accumulation and reduces mitochondrial stress. Thus, preimplantation embryos do employ stress response mechanisms to avoid the deleterious effects of PA exposure, but their protective ability may be overwhelmed by elevated PA levels. This information is vital to our understanding of the influence of obesity on human fertility. Investigating the extent of OA’s protective effects using different culture concentrations and times of exposure could contribute to optimizing culture environments for all preimplantation embryo culture systems, including that of the human.
